# Rapid Dynamics of Contrast Responses in the Cat Primary Visual Cortex

**DOI:** 10.1371/journal.pone.0025410

**Published:** 2011-10-05

**Authors:** Ming Hu, Yong Wang, Yi Wang

**Affiliations:** 1 State Key Laboratory of Brain and Cognitive Sciences, Institute of Biophysics, Chinese Academy of Sciences, Beijing, China; 2 Graduate University of Chinese Academy of Sciences, Beijing, China; Barrow Neurological Institute, United States of America

## Abstract

The visual information we receive during natural vision changes rapidly and continuously. The visual system must adapt to the spatiotemporal contents of the environment in order to efficiently process the dynamic signals. However, neuronal responses to luminance contrast are usually measured using drifting or stationary gratings presented for a prolonged duration. Since motion in our visual field is continuous, the signals received by the visual system contain an abundance of transient components in the contrast domain. Here using a modified reverse correlation method, we studied the properties of responses of neurons in the cat primary visual cortex to different contrasts of grating stimuli presented statically and transiently for 40 ms, and showed that neurons can effectively discriminate the rapidly changing contrasts. The change in the contrast response function (CRF) over time mainly consisted of an increment in contrast gain (CRF shifts to left) in the developing phase of temporal responses and a decrement in response gain (CRF shifts downward) in the decay phase. When the distribution range of stimulus contrasts was increased, neurons demonstrated decrement in contrast gain and response gain. Our results suggest that contrast gain control (contrast adaptation) and response gain control mechanisms are well established during the first tens of milliseconds after stimulus onset and may cooperatively mediate the rapid dynamic responses of visual cortical neurons to the continuously changing contrast. This fast contrast adaptation may play a role in detecting contrast contours in the context of visual scenes that are varying rapidly.

## Introduction

Due to the movements of objects, particularly those of eyes and head, incoming information to the visual system in natural vision is constantly varying [1]. For example, images projected on the fovea of the retina change three times per second owing to the saccadic eye movements [2]. Correspondingly, visual information falling on the receptive fields of cortical neurons is updated continuously. Consequently, local luminance and contrast in the receptive fields vary dramatically [3]. Furthermore, inputs to the visual system change transiently as a result of microsaccades which randomly move the eyes across a range of several dozen to several hundred photoreceptor widths [4] and evoke intense firing of neurons in the primary visual cortex (V1) by moving small receptive fields of the neurons across visual stimuli associated in the spatiotemporal context [5]. These abrupt changes of visual inputs have been thought to have significant impact on many aspects of visual processing, such as orientation tuning [6], [7], spatial frequency [8], and speed tuning [9]. It is thought that the visual cortex may adopt a rapid adaptive mechanism to match neural responses to the spatiotemporal contents of input stimuli [8], [10], [11].

In the contrast domain, it is well known that most V1 neurons show asymptotic saturation responses in which responses increase monotonically over a limited range of contrasts and saturate at high contrasts. After adapting to the prevailing contrast in an environment, neurons will shift the asymptotic (most sensitive) portion of their responses around this contrast level (contrast gain control) enabling them to more precisely distinguish ambient contrasts, particularly improving their discriminability to high contrasts that otherwise evoke the saturated responses. Contrast adaptation occurs in two different time scales, one is within 100 ms of contrast change [11], [12], and the other acting more slowly on a scale of 1∼10 s [13]–[17]. The former fast contrast adaptation has been explored mainly using stationary gratings with long blank intervals [18], [19]. However, the regular interstimulus interval of blanks used in the laboratory does not occur frequently in natural vision and could lead to biases in characterizing the properties of contrast responses (see Discussion). Although a few studies have investigated the mechanism responsible for fast contrast adaptation using contrast ramps without blanks [20], [21], it is not clear how contrast information is represented in visual cortical neurons when contrast changes transiently in a randomized order which resembles the contrast variations that often occur in normal vision [3], [22]. Exploring the dynamics of contrast responses under such conditions will enhance our understanding of the neural mechanisms for contrast contour processing in the context of natural visual scenes.

The present study was designed to examine the dynamics of contrast responses of neurons in the cat V1 in order to evaluate whether fast contrast adaptation works efficiently when stimuli change transiently. We used sinusoidal gratings at nine contrasts updated at 25 Hz in a random order and showed that V1 neurons are capable of discriminating contrasts under this stimulus protocol. Our results suggest that the fast adaptation mechanism to contrast is established by the cooperation of contrast gain control and response gain control immediately following stimulus onset.

## Results

**Figure 1:**
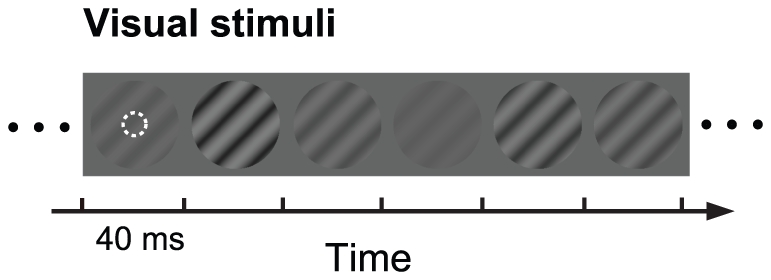
Stimulation paradigm. The stimulus set contained a series of 36 sinusoidal gratings having the same preferred spatial frequency and orientation, but having nine different contrasts and four spatial phases. All of them and the gray background had the same mean luminance (16.7 cd m^−2^). Each grating was presented for 40 ms. See the text for details. The dotted circle indicates the receptive field (RF) of a V1 neuron.

We measured responses of V1 neurons to different contrasts represented by serial gratings stimuli (Fig. 1). The complete set of stimuli containing 36 (9 contrasts×4 spatial phases) gratings were presented consecutively in a randomized order without blanks between successive gratings. Each grating in the sequence was lasted for 40 ms and repeated 200 times. In each repetition, the presentation sequence of the 36 stimuli was newly randomized. Thus each random sequence lasted for 1.44 s and the 200 repetitions took a total of 288 s (see Methods). We analyzed 23 simple cells and 78 complex cells from the V1 of 12 cats that had a signal/noise response ratio which met the criterion for data selection (Methods). Simple and complex cells behaved similarly in all the measurements described below, and are thus not stated separately in the following sections.

### Time course of contrast responses

**Figure 2:**
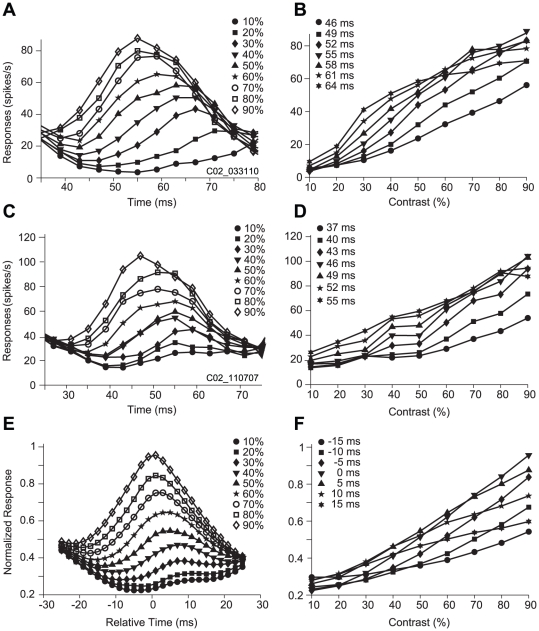
Time courses of responses of V1 neurons to rapidly changing contrasts. Responses were plotted as a function of time (left column) and contrast (right column). Panels *A*–*D* present two example cells. Panels *E* and *F* show the averaged response from a population of neurons (n = 101). *A*: Cell 1. Post-stimulus time histograms (PSTHs) were plotted for the 9 different contrast levels (different symbols). Each point is the averaged response that occurred within a 10-ms time window moving along the time axis in a step of 1 ms, here plotted every 4 ms for clarity. Each curve represents the responses to a single level of contrast. The responses were only plotted from 35 to 80 ms after stimulus onset for the clarity of viewing the changes that occurred during this time interval. *B*: The responses shown in *A* were plotted as a function of contrast at seven time points (different symbols) after the stimulus onset. *C* and *D*: Cell 2. *E* and *F*: Averaged data for the population of neurons. The conventions used are the same as those as in *A* and *B*. The PSTHs of each cell were normalized with their maximal response and aligned to their optimal latency (T_optimal_ of Fig. 3A; see Methods) before being averaged. The contrast response functions in *F* were plotted from PSTHs in *E* at seven time points.

Figure 2A shows responses of a neuron to nine levels of contrast over time. It can be seen that the change in temporal responses with contrast followed a regular manner, with the magnitude of responses decreasing and the latency of the maximal response increasing as contrast decreased. Figure 2B shows responses from the same cell as a function of contrast at seven time points during the course of the temporal responses. Responses increased almost monotonously with contrast in the rising phase of the PSTH (Post-Stimulus Time Histogram; 46 ms to 55 ms), but showed saturation at high contrast in the falling phase (55 ms to 64 ms). Thus the response profiles were different before and after the response peak. Figure 2C,D show the same measurements for another cell.

**Figure 3:**
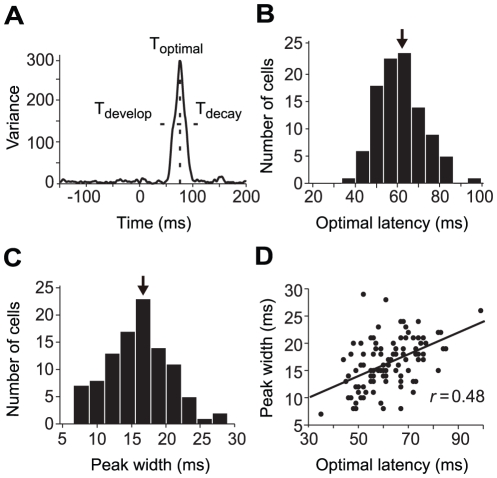
Temporal characteristics of the variance curve of responses to different contrasts. *A*: The variance curve of a typical cell. Optimal latency (T_optimal_) is given by the peak of the curve. Peak width of the curve was defined as the time difference between T_decay_ and T_develop_ at which the variance reached half of the peak magnitude. *B*: The distribution of the optimal latencies of a population of neurons (n = 101). *C*: The distribution of the peak width. In both histograms, the mean is indicated by an arrow. *D*: Scatter plot showing the significant correlation between the optimal latency and peak width of the variance curves.

We averaged PSTHs from a population of 101 cells (Fig. 2E). It is easily seen that the averaged response profile was similar to that of the single neurons (Fig. 2A,B). Figure 2F plots the averaged contrast response function for the population at seven time points evenly distributed around the optimal latency (0 ms in Fig. 2E; Fig. 3A). It is worth noting that there were clear vertical and horizontal shifts in the contrast response function between before and after the peak (e.g., −15 ms versus 15 ms; −10 ms versus 10 ms in Fig. 2F). This indicates that there were marked gain adjustments during the time course of responses. We note that responses to low contrast stimuli were below the mean firing rates (Fig. 1A,C,E). This was because in our stimulation paradigm with consecutive presentation of different contrasts there were no blank intervals between contrast stimuli and thus contrast adaptation took place. During the entire presentation, a total of 9×4×200 stimulations consisting of 9 different contrasts and 4 spatial phases (200 repetitions) were presented randomly in a test block lasting for 288 s, thus each stimulus contrast (having 4 spatial phases, see Methods) was preceded by the 9 contrasts in 800 times. On average the number of times a given contrast preceded the current stimulus was 88±11 (mean ± SD (hereafter for all data), n = 800 data points) across the population of 101 cells. The average contrast of the preceding stimuli was 50±25.8% (n = 800 data points). Given that the response to each contrast is the average adaptation to all the preceding contrasts, when the contrast of the current stimulus was lower than the average contrast, the average adaptation resulted in that the firing rate of neurons to the current stimulus was lower than the mean firing rate.

To evaluate the temporal characteristics of the gain adjustments of contrast responses under the fast presented stimulation, we considered three time points, T_optimal_, T_develop_, and T_decay_ (Fig. 3A, see Methods) from the variance curve of the temporal responses. We also defined the width of the peak response (peak width) as the time difference between T_decay_ and T_develop_ at half of the maximal magnitude of the variance curve. Figure 3B shows that T_optimal_ of the population of cells was distributed from 35 ms to 95 ms with a mean of 62±11 ms (n = 101). The peak width of the variance curve in the population of cells had a narrow distribution (Fig. 3C) with the mean peak width at 16±5 ms. Furthermore, there was a positive correlation (*r* = 0.48, *P*<0.001, n = 101) between optimal latency (T_optimal_) and peak width (Fig. 3D). This is similar to the result observed in a previous study [18].

### Dynamics of contrast response function

To quantitatively analyze gain adjustments during temporal responses, we fitted the contrast response function of neurons at T_optimal_, T_develop_ and T_decay_, respectively, using *Equation* (1). In the fits, *C_50_* was constrained to be no larger than 1 (100% contrast). Adjusted *R* square (ARS) values were computed to evaluate the goodness of fit (see Methods). The mean ARS of the population of neurons at these three time points were 0.91±0.11, 0.97±0.03, and 0.92±0.11 (n = 101), respectively, indicating that the fitted results can account for the original data beyond 90% on average and *Equation* (1) is an excellent fit for our data.

**Figure 4:**
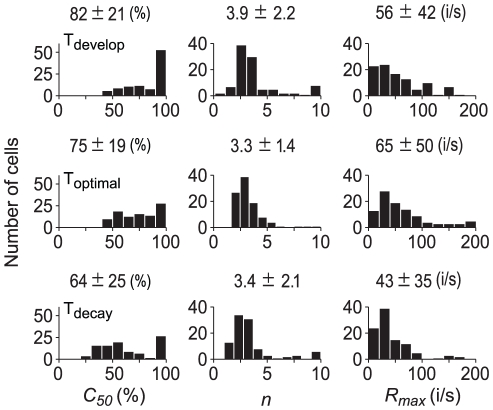
Population distributions of parameters of the contrast response function. The parameters, *C_50_*, *n*, and *R_max_*, are shown in the three columns, and the time points, T_develop_, T_optimal_ and T_decay_, are in three rows. The mean ± SD (n = 101 cells) is indicated at the top of each panel. %: % of contrast. i/s: spikes/s.

Figure 4 shows the distributions of *C_50_*, *n*, and *R_max_* at each of the time points for the population of neurons (n = 101). *C_50_* determines the contrast sensitivity of a neuron, the smaller value being associated with the higher contrast sensitivity to the lower contrasts. As seen in the first column of Figure 4, the *C_50_* mean decreased between T_develop_ and T_decay_ (*P*<0.01, one-way ANOVA). In addition, we note that the percentage of neurons with a *C_50_* between 90% and 100% was larger at T_develop_ (44%) than at T_optimal_ (21%) and T_decay_ (25%). These results suggest that there is a steady increase in the contrast sensitivity of neurons during the time course of responses, that is, the contrast response function shifts horizontally to the left over time (Fig. 2F). As for the exponent *n* (second column of Fig. 4), statistic analysis showed that there was no significant difference between the three time points (*P*>0.05, one-way ANOVA). The distributions of *R_max_* for the three time points are shown in the third column of Figure 4. There was significant reduction in *R_max_* between T_optimal_ and T_decay_ (*P*<0.01, one-way ANOVA), indicating a rapid decline in response after the peak. These data thus demonstrate that the changes observed in the time course of contrast responses of these neurons (Fig. 2F) might be due to the adjustment of contrast gain or response gain.

**Figure 5:**
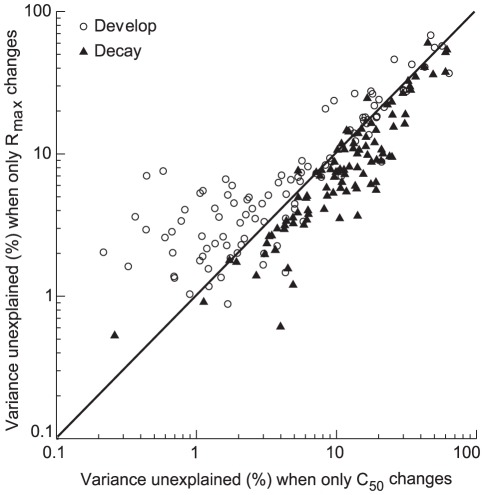
Scatter plot of the unexplained variance at the development phase and decay phase of contrast response function. Comparison of errors for the population of cells (n = 101) when contrast responses at T_develop_ (open circles) and T_decay_ (filled triangles) were fitted with *Equation* (1) in a single operation that allowed only the response gain (*R_max_*) or the contrast gain (*C_50_* ) to vary. The other parameters (except the *baseline*) which were not allowed to change were constrained to be the same as those at T_optimal_ for both curves. The least-squared fit error is given as the percentage of the variance of the data that is not accounted for, namely *R* square (see Methods) was multiplied by 100 to express the variance accounted for as a percentage of the total variation, thus the variance unexplained = 100−*R* square×100. The solid line is the diagonal line.

To further elucidate the mechanisms underlying these adjustments, we adopted a different fitting strategy [23]–[25]. Specifically, the contrast responses at T_develop_ and T_decay_ were fitted by holding *n* and *R_max_* constant at the same values as those for T_optimal_, allowing only *C_50_* free to change, or by holding *n* and *C_50_* constant at the same values as those for T_optimal_, allowing only *R_max_* free to change. The former fit corresponded to the adjustment of contrast gain, while the latter corresponded to the adjustment of response gain [24] when the contrast response functions at T_develop_ and T_decay_ were compared with those for T_optimal_. Figure 5 is a scatter plot comparing the least-squared fit errors between the two fits at T_develop_ and T_decay_. For most neurons, the unexplained variances were smaller when only *C_50_* changed at T_develop_ (open circles), while at T_decay_, the unexplained variances were smaller when only *R_max_* changes (filled triangles). The differences between the two fits at the two time points were both significant at the population level (Student's paired *t*-test, *P*<0.01, n = 101). These results suggest that the change in the contrast response function over time in the developing phase is more often an increment in contrast gain. However, a reduction in response gain plays a more important role in the decay phase.

### Contrast response and preferred spatial frequency

**Figure 6:**
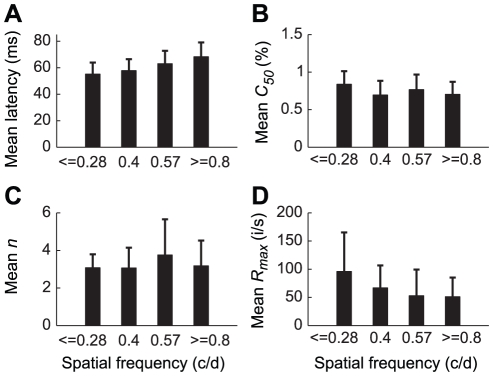
Relationship between the parameters of contrast response function and the preferred spatial frequency of cells. Cells were divided into four groups, based on their preferred spatial frequencies measured with the subspace reverse correlation methods (see Methods). The number of cells in each group was as follows: 22 (group 1, SF≤0.28), 22 (group 2, SF = 0.4), 24 (group 3, SF = 0.57), and 33 (group 4, SF≥0.8). The parameters of the contrast response function in each group are shown as mean ± SD (vertical bars and lines, note that these lines are not the s.e.m.). *A*: Optimal latency (Fig. 2B). *B: C_50_*. C: *n*. *D*: *R_max_*. c/d: cycles/degree. i/s: spikes/s.

In addition to analyzing dynamic responses, we also explored the relationship between the contrast response of neurons and the spatial frequency (SF) of stimuli. Neurons (n = 101) were divided according to their preferred spatial frequency into four groups from low to high SFs (Fig. 6). Figure 6 shows the relationships of T_optimal_, *n*, *C_50_* and *R_max_* of the contrast response function at T_optimal_ (Fig. 3B) with the preferred SFs of the neurons. First, there was an increase in T_optimal_ (Fig. 3A) with increasing preferred SF (Fig. 6A). One-way ANOVA showed that the relationship between response latency of the neurons and their SFs was significant (*P*<0.01). The difference of T_optimal_ between groups 1 (SF≤0.28) and 4 (SF≥0.8) and that between groups 2 (SF = 0.4) and 4 were significant (*P*<0.01). Second, there was no obvious relationship between mean *C_50_* and SFs (*P*>0.05, one-way ANOVA) though the mean *C_50_* in group 1 was significantly higher (*P*<0.05) than that of group 4 (Fig. 6B). Third, the mean *n* was not significantly different among the four groups (Fig. 6C). Fourth, mean *R_max_* decreased with increase of SFs and the mean *R_max_* in group 1 was significantly higher (*P*<0.05) than that of groups 3 and 4 (Fig. 6D), but the overall relationship between mean *R_max_* and SFs was not significant across the 4 groups of neurons (*P*>0.05, one-way ANOVA).

The results in Figure 6A suggest that the cells which prefer low SF process contrast information faster than those which prefer high SF (see Discussion). However, the results in Figure 6B,C,D reflect a random fluctuation in *C_50_*, *n*, and *R_max_* among the groups of cells having different preferred SFs, consistent with the previous finding that the distribution of contrast threshold is uniform across cells in the primary visual cortex [26], [27].

### Contrast response functions under different contrast ranges

The previous sections have shown that contrast gain and response gain are adjusted during the time course of responses. Next, we examined whether the contrast response function changes when the range of stimulus contrast varies. We adopted the same stimulus protocol as in the previous sections except that we used three contrast ranges: low (10% to 50%), medium (30% to 70%), and high (50% to 90%) in steps of 5% (see Methods). Contrast response functions were compared between the three contrast ranges as well as with the full range (10% to 90%).

**Figure 7:**
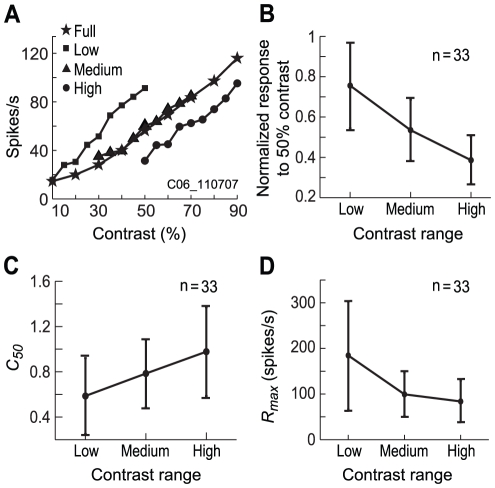
Contrast responses under stimulus contrasts distributing in different ranges. *A*: An example cell. Data from different ranges of contrast distributions were plotted with different symbols: □ **Full** for full range of contrast (10% to 90%), ▪ **Low** for low range (10% to 50%), ▴ **Medium** for medium range (30% to 70%), • **High** for high range (50% to 90%). *B*: The mean response of the 33 cells to 50% contrast decreased when the mean of the contrast range increased. The responses were normalized to the maximal response among the responses of each neuron to the 50% contrast contained in the three stimulus contrast ranges. *C*: The mean *C_50_* of the contrast response function increased with the increase in the mean of the contrast range when fitted with *Equation* (1) (holding *n* and *R_max_* constant). *D*: The mean *R_max_* of contrast response function decreased with the increase in the mean of the contrast range when fitted with *Equation* (1) (holding *n* and *C_50_* constant) (see Text for details). Note that the data presented in *B*, *C*, and *D* are the mean ± SD (n = 33), not the mean ± s.e.m.

Figure 7A shows the contrast response functions of a typical cell in the four conditions. The curves in the Figure exhibit two characteristics of gain adjustment. First, the contrast response function shifts horizontally along the contrast axis as the mean of the contrast range increases from low to high. Second, response magnitudes do not vary largely between the three different contrast ranges. These characteristics suggest that neurons adjust their responses according to the contrast range of a set of stimuli. We further analyzed this adjustment by extracting the responses of each neuron to 50% contrast from the three contrast stimulus sets and normalizing them to the maximal response obtained. The means (±SD) of the normalized responses to 50% contrast were then calculated in each contrast range for a population of neurons (n = 33). As Figure 7B shows, the mean of the normalized response to 50% contrast decreased significantly (*P*<0.01, one-way ANOVA) when the mean of the contrast range increased (0.76±0.21 for the low range, 0.53±0.15 for the medium range, and 0.39±0.12 (n = 33) for the high range). The relationship between the mean response and the mean stimulus contrast was analyzed using linear regression, and the slope obtained was −0.93, showing that the normalized response decreased by 0.93% as the contrast decreased by 1%. This result indicates that there is an obvious gain decrease when the mean of contrast range increases.

**Figure 8:**
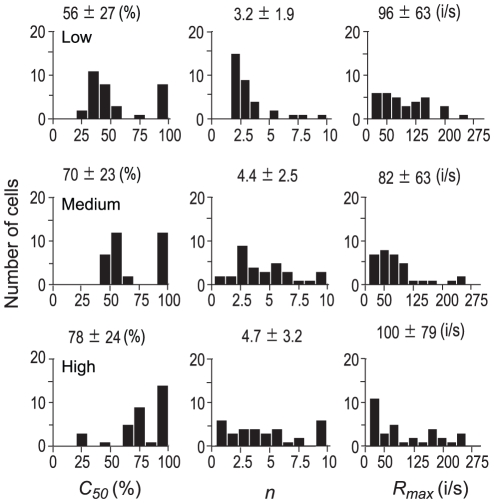
Distribution of the parameters of contrast response functions in different ranges of contrast distributions. Distributions of *C_50_*, *n*, and *R_max_* are shown in the three columns and the Low, Medium, and High contrast ranges are shown in the three rows. The mean ± SD (n = 33) is indicated at the top of each panel. %: % of contrast. i/s: spikes/s.

To further quantify this gain adjustment, the contrast responses obtained under the three contrast ranges was fitted with *Equation* (1). First, all four parameters in *Equation* (1) were allowed to vary. The mean ARS of the population of neurons in these three contrast ranges was 0.96±0.04, 0.94±0.05, and 0.91±0.07, respectively, showing a high quantitative fit to the sets of data. The distributions of *C_50_*, *n*, and *R_max_* in each of the contrast ranges for the population of neurons (n = 33) are shown in Figure 8. One-way ANOVA showed that there was a significant increase (*P*<0.01) in *C_50_* between the low and high contrast range. The difference in *n* or *R_max_* was not significant (*P*>0.05) between the contrast ranges. These results illustrate that contrast gain decreases with the increase of contrast range.

Next, we compared the fits produced by varying one of the four parameters (*R_max_*, *C_50_*, *n*, and *baseline*), while holding the other three parameters constant at the values obtained from the full range of stimulus contrasts. One-way ANOVA showed that the fraction of the variance explained (for its calculation in details, see the legend of Fig. 5) by allowing *R_max_* or *C_50_*, but not *n*, to vary was significantly larger (*P*<0.05, one-way ANOVA) than that obtained when the *baseline* was allowed to vary in the three ranges of contrasts (Table 1). Variance explained by varying *R_max_* or *C_50_* were also larger than that explained by allowing n to vary (though not statistically significant). Figure 7C shows the mean *C_50_* for the three contrast ranges. The mean *C_50_* significantly increased with the increase of contrast range (*P*<0.01, one-way ANOVA). The mean *C_50_* for the low contrast range was significantly smaller (*P*<0.01) than that for the high contrast range. The data suggest that the most sensitive range of contrast response function shifts to high contrasts when contrast range increases. Figure 7D displays the mean *R_max_* for the three contrast ranges. The mean *R_max_* significantly decreased with the increase of contrast range (*P*<0.01, one-way ANOVA). The mean *R_max_* for the low contrast range was significantly larger (*P*<0.01) than that for the medium and for high contrast ranges. The asymptotic shape of this curve illustrated that the adjustment of *R_max_* was nonlinearly compressed along the contrast axis at high contrast. These results indicate that adjustments in contrast gain or response gain underlie most of the differences in contrast response functions of the neurons in the three contrast ranges.

**Table 1:** The mean fractions of variances explained by fitting contrast response functions obtained in low, medium, and high ranges of contrast.

Parameters allowed to vary	*C_50_*	*R_max_*	*n*	*baseline*
**Low range (n = 33)**	93%	94%	79%	70%
**Medium range (n = 33)**	92%	94%	83%	80%
**High range (n = 33)**	89%	90%	70%	66%

## Discussion

The purpose of the present study was to address how rapidly changing luminance contrast is represented in the cat primary visual cortex. Using contrast stimuli presented randomly at 25 Hz without a blank interval between successive stimuli, we have shown that (1) the increment in contrast gain mediates the development of the contrast response function, while the decrement in response gain played a more important role in the decay phase; (2) the increase in the mean of contrast range decreases the contrast gain and the response gain of a neuron. These results indicate that neurons in the cat primary visual cortex can detect and discriminate contrasts varying as rapidly as 25 Hz and that the contrast sensitivity of a neuron changes with different contrast ranges in a dynamic fashion.

### Contrast sensitivity of neurons depends on the stimulus paradigms

The parameter of *C_50_*, semisaturation constant, of contrast response function is an indication of the sensitivity of a neuron in response to contrast. The *C_50_* is the contrast that requires for evoking 50% of the maximal response (*R_max_*) and corresponds to the steepest point of contrast response function. In other words, a neuron is most sensitive to the contrast change around *C_50_*. The neurons having a smaller *C_50_* are more sensitive to the contrast change at the lower levels and show more saturated responses to the higher contrasts, while those having a larger *C_50_* are more sensitive to the contrast change at the higher levels or demonstrate a more linear relationship between the response magnitude and the contrast magnitude [15], [16], [23]. Under the condition of the transient contrast stimulation we used, many neurons showed a large *C_50_* (Fig. 4). In our experiment, the percentage of neurons with *C_50_*>90% (*C_50_* was constrained to be no larger than 1 in the fit) was 44% at T_develop_, 21% at T_optimal_, and 25% at T_decay_, suggesting that more neurons possess linear contrast response function in the development phase than the later stage of temporal responses to contrasts (Fig. 2F). Furthermore, all these percentages are higher than that reported by previous studies [18], [23]. In Albrecht et al.'s study [23], in which the stimuli consisted of 20 cycles of optimized drifting gratings followed by 15 s of no-pattern luminance blank, this percentage was 9%, while it was approximately 5% when Albrecht et al. used stationary grating patterns flashed for 200 ms with a 300 ms interval blank of mean luminance [18]. This difference in the percentages between our results and the previous results suggests that contrast sensitivity of neurons changes under different stimulus conditions. The no-pattern mean luminance adopted in Albrecht et al.'s experiments was to minimize the interactions between successive grating patterns. However, neurons may adapt to the mean luminance (0% contrast) during the interval between contrast patterns and increase their contrast sensitivity because grating patterns with a contrast as low as 3% enhance the contrast sensitivity of V1 neurons [13]. Moreover, low contrast grating patterns that evoke few spikes cause substantial hyperpolarization [28]. Hyperpolarization recovers neuronal sensitivity to a relatively high contrast. Thus, when tested with contrast patterns after a low contrast stimulus or a blank having the same mean luminance, the contrast response function is expected to have a lower *C_50_* value.

### Shift in optimal latency of contrast response function

It is known that the preferred spatial frequency of a V1 neuron shifts from low to high over time [29]–[31]. Frazor et al. [30] showed that the mean rate of this shift is approximately 0.05 octave ms^−1^, that is, response latency increases at a mean rate of 20 ms octave^−1^ with spatial frequency. These results were obtained by using gratings with a specific contrast. Here we found that latency of the entire contrast response function also increases with spatial frequency at the population level (Fig. 6A). The mean rate of this increase in our sample of neurons was 22 ms octave^−1^ (obtained by linear regression of the data in Fig. 6A) which is similar to the result obtained by Frazor et al. [30].

Furthermore, at a specific spatial frequency, the latency of neural responses increased when contrast decreased [32], [33]. Albrecht et al. [18] found that the mean latency shift between the highest contrast and the lowest contrast was 65.3 ms. However, the shift in our population data was much shorter, approximately 10 ms (Fig. 2E). This suggests that V1 neurons may have faster temporal dynamic under transient stimulation in comparison to the steady-state condition [20], [24]. This conclusion is also consistent with recent findings that the timescale of neuronal responses is subject to change correlating with the spatial and temporal contexts of visual stimuli [34], [35].

### Contrast gain control and response gain control

Previous studies have shown that there are two different mechanisms, contrast gain control and response gain control, which mediate the change in contrast response over time. The former mechanism is adopted in normalization models [36]–[38], while the latter is adopted in synaptic models [24], [39]. Normalization models can account for several contrast-dependent properties of cortical neurons, such as size tuning, cross-orientation inhibition, and response saturation. Synaptic models provide a good interpretation of the rapid decay of the high initial discharge rate in transient responses to a stationary stimulus. However, Müller et al. [24] showed that it is usually a reduction in response gain rather than a reduction in contrast gain that mediates the change over time between contrast response functions measured early (first 100 ms) and late (500 ms after stimulus onset) in the temporal response. Moreover, Müller et al. [24] showed that response saturation is more common in the later stages of responses. These results seem to contradict the normalization model while favoring the synaptic model.

Our findings provide a plausible explanation for this contradiction. As shown in Figure 5, the developing phase of the contrast response function mainly exhibits an increment in contrast gain, while the decay phase of the contrast response function mainly exhibits a decrement in response gain. Thus we propose that, when visual stimulation is changing rapidly, contrast gain control may work in a rapid manner [21], while response gain control may act relatively slowly. The former operates by rapidly adjusting gain and integration time according to local luminance and contrast signals [3], [40], while the latter improves stimulus coding by shifting the gain of cortical circuits over time [41]. These two mechanisms may work cooperatively in mediating contrast-dependent lateral connectivity [42] and further contribute to the short-term enhancement of synaptic effectiveness [35], [43] which has been thought to be the neurophysiological correlate of sensory perception.

On the other hand, our study also shows that the contrast response function shifts horizontally with the distribution range of the rapidly changing contrasts (Figs. 7,8). This is consistent with results from previous studies using prolonged presentation of contrasts [13]–[17], implying that the slow contrast adaptation (1∼10 s) found in previous studies [13]–[17] also occurs in our stimulation paradigm. Since a set of stimuli from a certain range of contrasts was repeated every 1.44 s in the current experiment, this kind of slow contrast adaptation must accumulate from the beginning to the end of the stimulation protocol, but would become stable after the first couple of repetitions of the set stimulus presentation since there should be a steady state of adaptation during normal vision. The slow contrast adaptation might act relatively independently [3] from the fast contrast adaptation we have described here. The detailed differences or relationships between the two adaptation processes remain to be explored further.

Several studies have found that contrast adaptation occurs in LGN neurons under both artificial stimuli and natural scene movies [3], [40], [44]. Furthermore, models that incorporate the mechanism of fast gain control are powerful in predicting responses of LGN neurons to natural scenes [45]. Thus, it would be intriguing to see if V1 neurons demonstrate properties similar to those we have described here under natural scene stimulation. Studying the contrast response of V1 neurons to natural scene stimulation is crucial for understanding normal vision, since our eyes are never still, even in scanning a scene when gazing at an interesting target during free viewing [4]. Fixational eye movements occur over tens of milliseconds [4], a timescale similar to the 40 ms of the stimulus presentation used here.

In conclusion, our results suggest that V1 neurons efficiently distinguish prevailing contrasts in the environment with the most sensitive portion of the contrast response function by the mechanism of fast contrast adaptation. The processes could occur on a timescale as short as 40 ms by rapid adjustments in both contrast gain and response gain in which contrast gain control might play a more major role in the processes. This study has contributed to our understanding of the mechanism of contrast processing in the primary visual cortex that occurs in the context of rapid change in the visual scenes.

## Materials and Methods

### Physiological preparation

Twelve normal adult cats (1.5–3 kg) were prepared for extracellular recording. The protocols were described in detail elsewhere [46], [47] and are briefly stated here. The trachea and forelimb vein were first cannulated after intramuscular administration of ketamine (20 mg kg^−1^). Surgery was performed under deep intravenous anesthesia using a combination of propofol and sufentanil. An approximately 2.5 mm×2.5 mm craniotomy centered at Horsley-Clarke coordinates P 2.5 mm and L 2.5 mm was made to access cells representing the central visual field in the primary visual cortex (area 17). Anesthesia was maintained throughout the duration of the recording experiment with infusion of propofol (1.8–2.2 mg kg^−1^ h^−1^, i.v.) and sufentanil (0.15–0.22 µg kg^−1^ h^−1^, i.v.), and paralysis was maintained with gallamine triethiodide (10 mg kg^−1^ h^−1^, i.v.). The physiological state of the animal was monitored by body temperature (38°C), end-tidal CO_2_ (approximately 4.2%), ECG (approximately 200 beats/min), and EEG to estimate the depth of anesthesia. This was also judged by regular testing for responses of the animal to toe or ear pinching while monitoring heart rate changes. The pupils were dilated by local administration of homatropine and the nictitating membranes were retracted with phenylephrine hydrochloride. Contact lenses of sufficient power and 3 mm artificial pupils were placed on the corneas to focus the eyes on a CRT monitor 57 cm away. Glass-coated tungsten microelectrodes (impedances of 1 to 3 MΩ) were inserted into the cortex and driven by a microelectrode driver (Narishige). Extracellular potentials of cells driven by stimulating the receptive field (RF) of the dominant eye were isolated, amplified, and filtered, then sampled at 12 kHz and saved with a TDT RA16 interface and OpenEX software (Tucker-Davis Technologies, Inc., USA). Individual units were further identified with TDT OpenSorter offline. All animal care and experimental procedures were approved by the Institutional Animal Care and Usage Committee (IACUC) of the Institute of Biophysics, Chinese Academy of Sciences (ID: SYXK(PTJ)2008-114) and followed the guidelines of the National Institutes of Health (USA). Experiments were designed to minimize suffering and the number of animals used.

### Preliminary measurements

Visual stimuli were displayed on a cathode-ray-tube (CRT) monitor (Iiyama HM204DT A, at a resolution of 800×600 pixels and a refresh rate of 100 Hz) at a viewing distance of 57 cm. The monitor was calibrated to obtain a precise match between the requested and actual luminance. We conducted the following preliminary measurements once the action potentials of cell units were isolated. First, the approximate position and size of the receptive field, the preferred orientation, spatial frequency, and temporal frequency were qualitatively determined by manually varying the stimuli in these dimensions while listening to the firing rate of the units. Second, the orientation and spatial frequency tuning of the recorded cell were quantitatively measured with subspace reverse correlation methods [31], [48], [49]. Third, a standard reverse correlation procedure [50] was performed to obtain the accurate position and spatiotemporal organization of the receptive field. Fourth, gratings positioned at the preferred orientation and spatial frequency but drifting at a range of temporal frequency in two directions perpendicular to the preferred orientation were used to determine the temporal frequency tuning. All the gratings had a Michelson contrast of 60%. Responses of cells to the preferred temporal frequency and direction were used to calculate the F_1_/F_0_ modulation ratio according to the criteria described in [51].

### Visual stimuli

To measure contrast responses of a cell, a set of sinusoidal gratings of the preferred spatial frequency and orientation but of different contrasts and spatial phases was generated. Contrast ranged from 10% to 90% in 10% steps. For each contrast, four spatial phases (0°, 90°, 180°, 270°) of a grating, a quarter of a cycle of the preferred spatial frequency (360°), were included. A complete set of contrast stimuli contained 36 (9×4) gratings. These gratings were presented continuously one after another in a randomized order, each of them lasting for 40 ms (4 video frames) on a uniform background with the same mean luminance of 16.7 cd m^−2^ as that of the gratings (measured by ColorCAL colorimeter, Cambridge Research System, Ltd). There were no blanks between any consecutive grating stimuli. Therefore, one presentation of the set of stimuli took 1.44 s. Each stimulus was repeated 200 times to accumulate sufficient spikes and the entire stimulus presentation in a block lasted for a total of 288 s. The stimuli presented here were in a pseudorandom sequence using the reverse correlation methods [31], [48], [49]. Thus, each contrast was preceded 800 times (4 spatial phases×200 repetitions) by all nine contrasts. The diameter of the gratings was three times larger than the largest dimension of the conventional receptive field of the cell being recorded.

To examine the adaptation to different ranges of contrast changes, a portion of the cells (n = 33) were also tested with the other three ranges of stimulation contrasts. The low range of contrasts was from 10% to 50% in 5% steps, the medium range was from 30% to 70% in 5% steps, and the high range was from 50% to 90% in 5% steps.

### Data analyses

To calculate average temporal responses to stimuli in a range of contrasts, stimulus-triggered averages were calculated for each contrast in a stimulus sequence as follows [52]. Each time a given contrast appeared in the stimulus sequence, spikes in the following 200 ms after stimulus onset were counted at a 1 ms resolution and one such stimulus presentation was regarded as a trial. Responses to the 4 spatial phases of a grating stimulus for a given contrast were assigned to the responses to that contrast to remove effects of different spatial phases of a grating stimulus on responses of a neuron. For each contrast, spikes were summed across all such trials (800 = 4 spatial phases×200 repetitions/contrast) to obtain the averaged temporal response over the 200 ms period. Then, the data were smoothed with a 10 ms width of Gaussian window in a step of 1 ms.

Variance was calculated across all stimulus contrasts in a set as a function of time. To determine whether a cell would be included in the further analyses, the mean and SD of the noise responses were calculated from the variances in the 150 ms preceding the stimulus onset, and the cell was accepted only if its variance reached a peak that exceeded 5 SDs higher than the mean of the noise. We used the variance curve (the time course of the variance; e.g., Fig. 3A) to define three time points at which the contrast response functions were subsequently analyzed. These corresponded to the time point at which the variance achieved its maximum value (optimal latency, T_optimal_; Fig. 3A, vertical dashed line), and to the other two time points at which the variance reached half of the maximal value during the development (T_develop_) and decay (T_decay_) phases of the variance curve. The peak width of the variance curve was defined as the time difference between T_decay_ and T_develop_. The contrast response functions of each cell were taken at these time points from its averaged temporal responses.

To quantify and compare the properties of the contrast response functions at these time points, the Naka-Rushton equation [15], [18], [23], [53], [54] was used to fit the contrast responses:
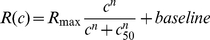
(1)where *R* and *c* are the responses and the contrast, *R_max_* is the maximal response to the contrast after subtracting the *baseline*, the maintained discharge; *n* and *c*_50_ are the parameters that define the steepest slope of the contrast response function and the contrast at which the steepest portion is centered, that is, the contrast at which 50% of *R_max_* is evoked.

To assess goodness of fit, adjusted *R* square (ARS) was calculated to quantify the variations in the data that were accounted for, using the following standard procedure. First, we calculated the variance of the data (“total variation”). Second, we calculated the sum of the squared deviations between the data and the fitted results (“residual variation”). Third, we calculated “*R* square” by subtracting the residual variation from the total variation and dividing the result by the total variation. Finally, the *R* square was adjusted as follow:
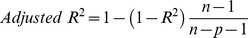
(2)where *n* is the number of observations (n = 9), and *p* is the number of independent variables. This adjustment was used to evaluate the fitting effciency when the number of indpendent variables changed. An ARS close to 1 indicates an excellent fit for the data.
